# Efficacy and Safety of Stem Cell Therapy in Children With Autism Spectrum Disorders: A Systematic Review and Meta-Analysis

**DOI:** 10.3389/fped.2022.897398

**Published:** 2022-05-04

**Authors:** Jiayang Qu, Zicai Liu, Lincai Li, Zhengwei Zou, Zhengyi He, Lin Zhou, Yaolin Luo, Minhong Zhang, Junsong Ye

**Affiliations:** ^1^The First Clinical Medicine College of Gannan Medical University, Ganzhou, China; ^2^Subcenter for Stem Cell Clinical Translation, First Affiliated Hospital of Gannan Medical University, Ganzhou, China; ^3^School of Rehabilitation Medicine Gannan Medical University, GanZhou, China; ^4^Ganzhou Key Laboratory of Stem Cell and Regenerative Medicine, GanZhou, China; ^5^Clinical Medicine Research Center, First Affiliated Hospital of Gannan Medical University, Ganzhou, China; ^6^Key Laboratory of Prevention and Treatment of Cardiovascular and Cerebrovascular Diseases, Ministry of Education, Gannan Medical University, Ganzhou, China; ^7^Key Laboratory of Biomaterials and Biofabrication in Tissue Engineering of Jiangxi Province, Gannan Medical University, Ganzhou, China

**Keywords:** autism spectrum disorders (ASD), stem cell therapy, meta-analysis, efficacy, safety

## Abstract

**Aim:**

There is insufficient evidence regarding the efficacy and safety of stem cell therapy for autism spectrum disorders. We performed the first meta-analysis of stem cell therapy for autism spectrum disorders in children to provide evidence for clinical rehabilitation.

**Methods:**

The data source includes PubMed/Medline, Web of Science, EMBASE, Cochrane Library and China Academic Journal, from inception to 24th JULY 2021. After sifting through the literature, the Cochrane tool was applied to assess the risk of bias. Finally, we extracted data from these studies and calculated pooled efficacy and safety.

**Results:**

5 studies that met the inclusion criteria were included in current analysis. Meta-analysis was performed using rehabilitation therapy as the reference standard. Data showed that the Childhood Autism Rating Scale score of stem cell group was striking lower than the control group (WMD: −5.96; 95%CI [−8.87, −3.06]; *p* < 0.0001). The Clinical Global Impression score consolidated effect size RR = 1.01, 95%CI [0.87, 1.18], Z = 0.14 (*p* = 0.89), the effective rate for The Clinical Global Impression was 62% and 60% in the stem cell group and the control group, respectively. The occurrence events of adverse reactions in each group (RR = 1.55; 95%CI = 0.60 to 3.98; *p* = 0.36), there was no significant difference in the incidence of adverse reactions between the stem cell group and the control group.

**Conclusions:**

The results of this meta-analysis suggested that stem cell therapy for children with autism might be safe and effective. However, the evidence was compromised by the limitations in current study size, lacking standardized injection routes and doses of stem cells, as well as shortages in diagnostic tools and long period follow-up studies. Hence, it calls for more studies to systematically confirm the efficacy and safety of stem cell therapy for children with autism spectrum disorders.

## Introduction

Autism spectrum disorder (ASD) is a group of neurodevelopmental disorders characterized by social deficits, communication inabilities and stereotypic behaviors (1). The incidence is estimated to be 1–2% of all children, according to the U.S. Centers for Disease Control and Prevention (2). Despite its increasing prevalence (3, 4), the etiology of ASD is not fully understood yet, which can be interpreted as including both genetic and environmental factors (5). The processes of inflammation and immune activation may act to modify the risk of ASD gene expression or destruct process of typical neural development in ASD (6). ASD patients are a heterogeneous group with different symptom characteristics (7), thus there is no definitive treatment for ASD patients (8).

Given the potential effects of sustained immune disorders and inflammation in ASD and known paracrine (9), homing (10, 11), immunomodulatory (12) and multi-directional differentiation capacity (13, 14) of stem cells, they are receiving attention as a potential therapeutic approach. Growing numbers of research reports have confirmed the efficacy and safety of stem cell therapy with different methods including autologous bone marrow mononuclear cells (15–17), fetal stem cells (18), human cord blood mononuclear cells (19) and umbilical cord mesenchymal stem cells (20) in patients with autism. However, the great mass of these case reports or case series studies are limited to a few geographical regions, thus fail to provide sufficient guidelines for clinical decisions. Moreover, these studies have several shortcomings, such as small sample size, non-standard control groups, non-standard assessment scale and short-term follow-up. Most importantly, when we began this study, there was no systematic evidence-based medical review demonstrating the efficacy and safety of stem cell therapy for ASD.

Collectively, we aim to rigorously screen and extract all preclinical trial data on stem cell therapy for autism, objectively evaluate and summarize evidence concerning the effectiveness of stem cell therapy for autism symptoms through systematic review and meta-analysis.

## Methods

The study design was developed by the steering group, followed by the standard Cochrane Neonatal Review Group methods and PRISMA reporting guidelines. We have already submitted a registration application at Prospero (CRD42021285384, https://www.crd.york.ac.uk/prospero/).

### Population

Children and adolescents (age 0–18 years) were diagnosed with autism, regardless of region, gender, or race.

### Intervention

Multiple kinds of stem cells interventions on children with autism were investigated within the current systematic review and meta-analysis, with no limitations on injection times, administration route and dose. Studies of stem cells in combination with other treatments, such as antipsychotic drugs, are also being considered.

### Comparisons

Rehabilitation therapy includes sensory integration therapy, auditory training, behavioral intervention, occupational therapy, speech therapy and music therapy.

### Outcomes

The main indicators are the scores of Clinical Global Impression (CGI) and Childhood Autism Rating Scale (CARS) or any other evaluation tools suitable for ASD in the corresponding studies.

### Study Types

Randomized controlled trials (RCTs) and controlled clinical trials (CCTs) were included in this study, both paralleled or crossover. For trials that had a crossover design, we included all the data before and after the crossover.

### Data Sources

The following English and Chinese databases were searched from their inception to 24th JULY 2021: PubMed/Medline, Web of Science, EMBASE, Cochrane library, China Academic Journal (through China National Knowledge Infrastructure [CNKI], [WanfangData], [Cqvip], [SinoMed]) and Clinicaltrials.gov. A detailed illustration of search strategies is available in Supporting information 1 (S1. Search Strategy). No date restrictions or language restrictions are used for retrieval. Finally, references were tracked and included in the study to ensure that no RCTs and CCTs were missed by the search strategy.

### Study Selection

All prospective controlled clinical studies of stem cell therapy on autism patients were included, as were trials in which stem cells were part of a complex intervention. We excluded qualitative studies, uncontrolled trials, case studies and case series, as well as trials that developed between different cell types, and studies that failed to provide detailed results.

### Data Extraction and Quality and Validity Assessment

Two independent reviewers evaluated the retrieved studies for inclusion and assessed the methodological quality of included studies. Elements extracted included study characteristics (author, country, publication year and experimental design), participant characteristics (sex, age range and diagnostic criteria), intervention details (types of cells, dose ranges, administration and frequency), outcome measurement, follow-up time and adverse reactions. The risk of bias was assessed using the Review Manager 5.3. The disagreements were thrashed out by the two reviewers.

### Data Analysis

Data entry and analysis were performed using Review Manager 5.3 software. The data required for meta-analysis were obtained by direct input or indirect calculation based on the original data (The data of CARS can be obtained directly, and the effective improvement number of CGI needs to be calculated based on the total efficiency provided in the original text multiplied by the number of each group, like Dawson *et al*.'s study). When studies of multiple intervention groups are compared, the “shared” control group is split equally in each comparison (21) and the weighted average difference (WMD) and risk ratio (RR) were used to compare continuous variables (CGI and CARS) and dichotomous variables (Adverse events) respectively. All results obtained were reported with 95% confidence intervals (CI). Heterogeneity among studies was determined by Q test and I^2^ statistics (I^2^ equals or exceeds 50%, *p* < 0.05 is considered to have greater heterogeneity). The random effect model or mixed effect model was selected according to the size of heterogeneity. With high heterogeneity, sensitivity analysis or subgroup analysis was used to detect the source of heterogeneity; if the source of heterogeneity cannot be found, a descriptive analysis was conducted.

## Results

### Results of the Search

**Figure 1:**
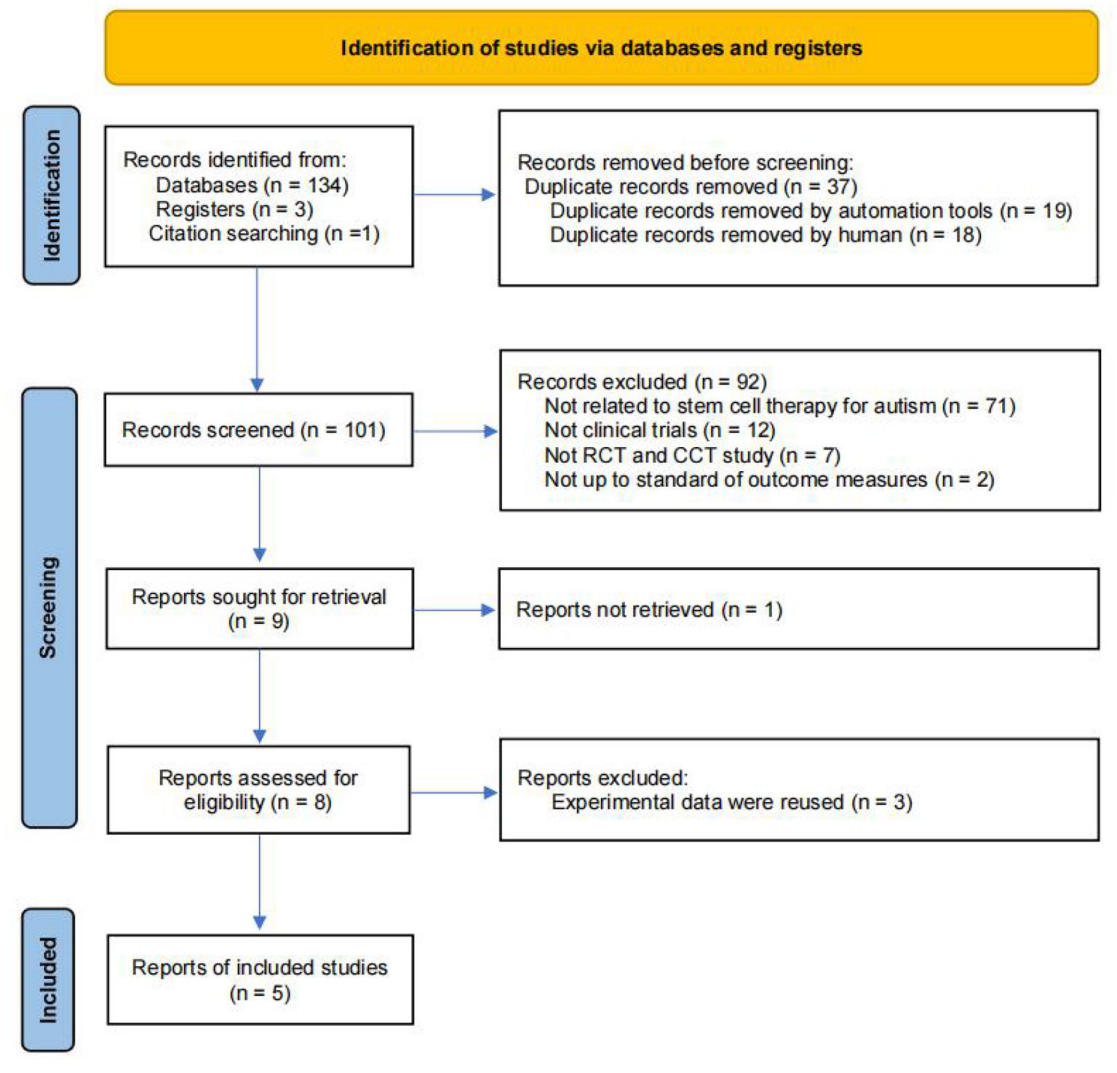
The inclusion flow chart of the literature was retrieved.

A flowchart describing the selection of eligible trials is presented in Figure 1. A total of 137 articles from 9 databases were retrieved: Web of Science (*n* = 15) databases, PubMed/MEDLINE (*n* = 12), Cochrane (*n* = 7), Embase (*n* = 36), CNKI (*n* = 11), Wan fang Data (*n* = 30), Cqvip (*n* = 19), Sino Med (*n* = 4), Clinicaltrials.gov (*n* = 3). We also included 1 latest research reports through citation searching to ensure that the retrieved literature includes all the studies in the published meta-analysis (26). Finally, 5 studies were included within our meta-analysis.

### Characteristics of the Studies

The characteristics of the included studies are listed in Table 1. Diagnosis criteria were performed mainly through the Diagnostic and Statistical Manual of Mental Disorders-4 (DSM-4) (60.0%) or Diagnostic and Statistical Manual of Mental Disorders-5 (DSM-5) (40.0%) criteria. The 5 studies comprised 3 RCTs and 2 CCTs, sample sizes ranged from 36 to 180. A total of 325 subjects were included in the systematic review and meta-analysis, 319 of whom were analyzed for safety, including 265 males and 54 females. However, in Dawson's study (27), two pilot participants and 2 participants were found to be ineligible [bipolar disorder (*n* = 1), the primary caregiver does not speak English (*n* = 1)] after randomization, thus were excluded from the efficacy analysis. Collectively, only 315 subjects were included in the efficacy analysis.

**Table 1:** Summary of clinical studies of stem cells therapy for autism spectrum disorders.

**Study**	**Country**	**Design**	**Sample size**		**Age, y**	**Diagnostic criteria**	**Therapy**			**Control**		**Follow up**	**Main outcome measures**	**Adverse reaction**
			**(aggregate/ analyzed)**	**(male/** **female)**			**Intervention**	**Administration**	**Sample size**	**Control intervention**	**Sample size**			
Sharifzadeh et al. (22)	Iran	RCT	36/32	27/5	5-15	DSM-5	BMMSC, first,0.5-1 × 10^8^ cells per 2.0 mL.s,0.3-0.5 × 10^8^per 2.0 mL.	Intrathecally injection.	14	Rehabilitation therapy and risperidone	18	6 and 12 M	CARS, GARS-II, CGI	In general, injection-related side effects of 12 months were not observed in any of the patients.
Liu (23)	China	CCT	42/42	35/7	3–12	DSM-4	Liu 2010 a: MNCs, cells per serving was 2 × 10^6^/kg.	Intravenous infusion once, lumbar puncture subcobweb space injection 3 times.	14	Rehabilitation therapy	14	4W,4M	CARS, CGI	There were 12 cases of low fever, 4 cases of headache, 3 cases of lumbago, 4 cases of fatigue, and 2 cases of nausea and vomiting, without serious adverse reactions.
							Liu 2010 b:MNCs and MSCs. Cells per serving was 2 × 10^6^/kg.	Intravenous infusion once, lumbar puncture subcobweb space injection 3 times.	14					
Dawson et al. (24)	USA	RCT	180/176	143/37	2–7	DSM-5	CB, the number of therapeutic cells≥2.5 × 10^7^cells/kg.	Peripheral intravenous infusion.	119	The placebo (TC199 + 1% DMSO) and behavioral intervention	61	24W	VABS-3, PDDBI, CGI-S, CGI-I, and EOWPVT-4	3 in the placebo arm, 1 in the autologous CB cohort,and 2 in the allogenic CB cohort. None of these events was related to the study product.
Lv et al. (19)	China	CCT	37/36	35/1	3–12	DSM-4	Lv 2013a: CBMNC and UCMSCs,cells per serving was 2 × 10^6^/kg.	Once intravenous and three times intrathecal infusions.	9	Rehabilitation therapy	13	4W, 8W, 16W, 24W	CARS,ABC,CGI	No significant safety issues related to the treatment and no observed severe adverse effects.
							Lv 2013b: CBMNC cells per serving was 2 × 10^6^/kg.	Once intravenous infusion and three times intrathecal injections.	14					
Chez et al. (25)	USA	RCT	30/29	25/4	2–6	DSM-4	AUCB, at least 10 × 10^6^/kg, 50 ml.	Peripheral intravenous infusion.	29	Infusion of 0.9% saline placebo	29	12W, 24W, 37W, 49W	EOWPVT-4,ROWPVT-4,Stanford Binet,CGI	No adverse events required treatment. There were no observed allergic reactions or serious adverse events associated with the administration of AUCB.

*RCT, randomized controlled trial; CCT, controlled clinical trail; DSM,Diagnostic and Statistical Manual of Mental Disorders; BMMSC, bone marrow mesenchymal stem cell; MNCs, mononuclear cells; MSCs, mesenchymal stem cells; CB, cord blood; UCMSCs, umbilical cord mesenchymal stem cells; AUCB,autologous umbilical cord blood; DMSO, Dimethyl sulfoxide; CARS, childhood autism rating scale; CGI, Clinical Global Impression; VABS, Vineland Adaptive Behavior Scales; PDDBI, The PDD Behavior Inventory; EOWPVT, Expressive One-Word Picture Vocabulary Test; ABC, Autism Behavior Checklist; ROWPVT, receptive one word picture vocabulary test; M, month; W, week*.

### Methodological Quality

**Figure 2:**
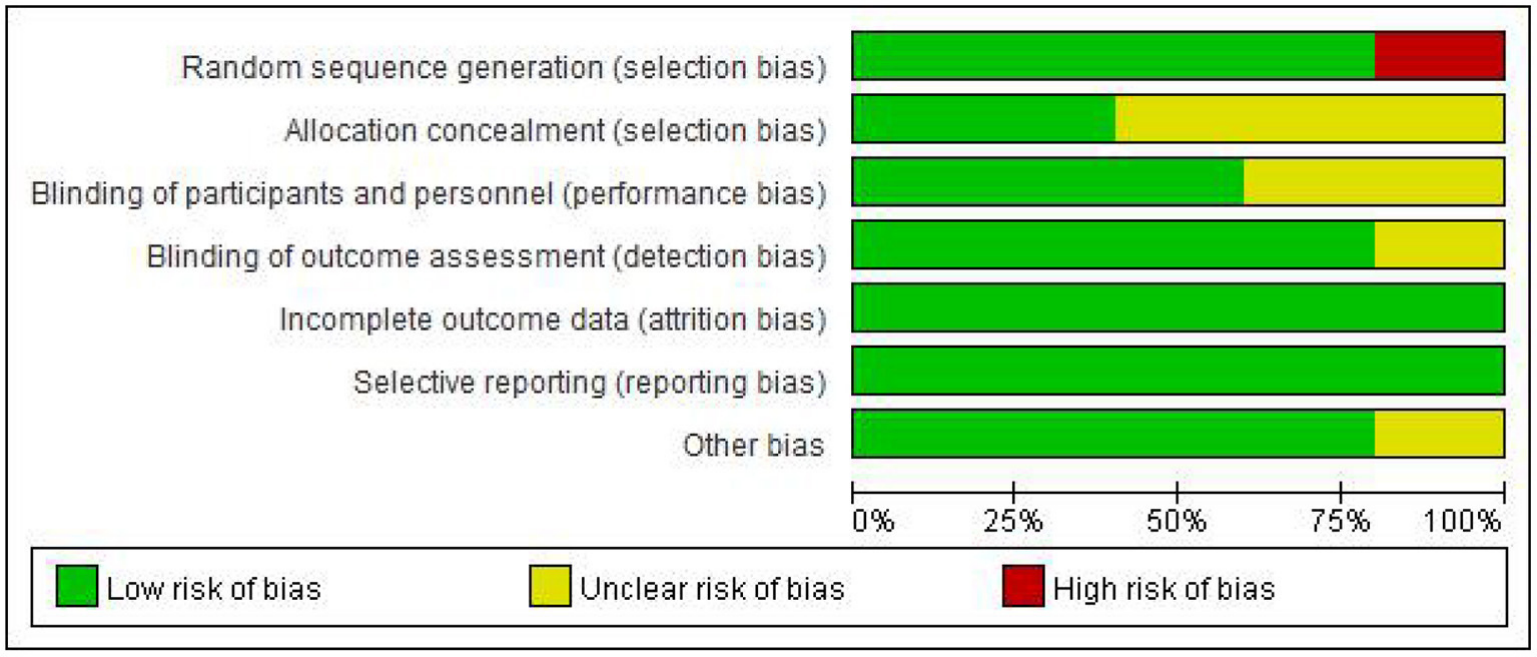
Risk of bias and applicability concerns.

**Figure 3:**
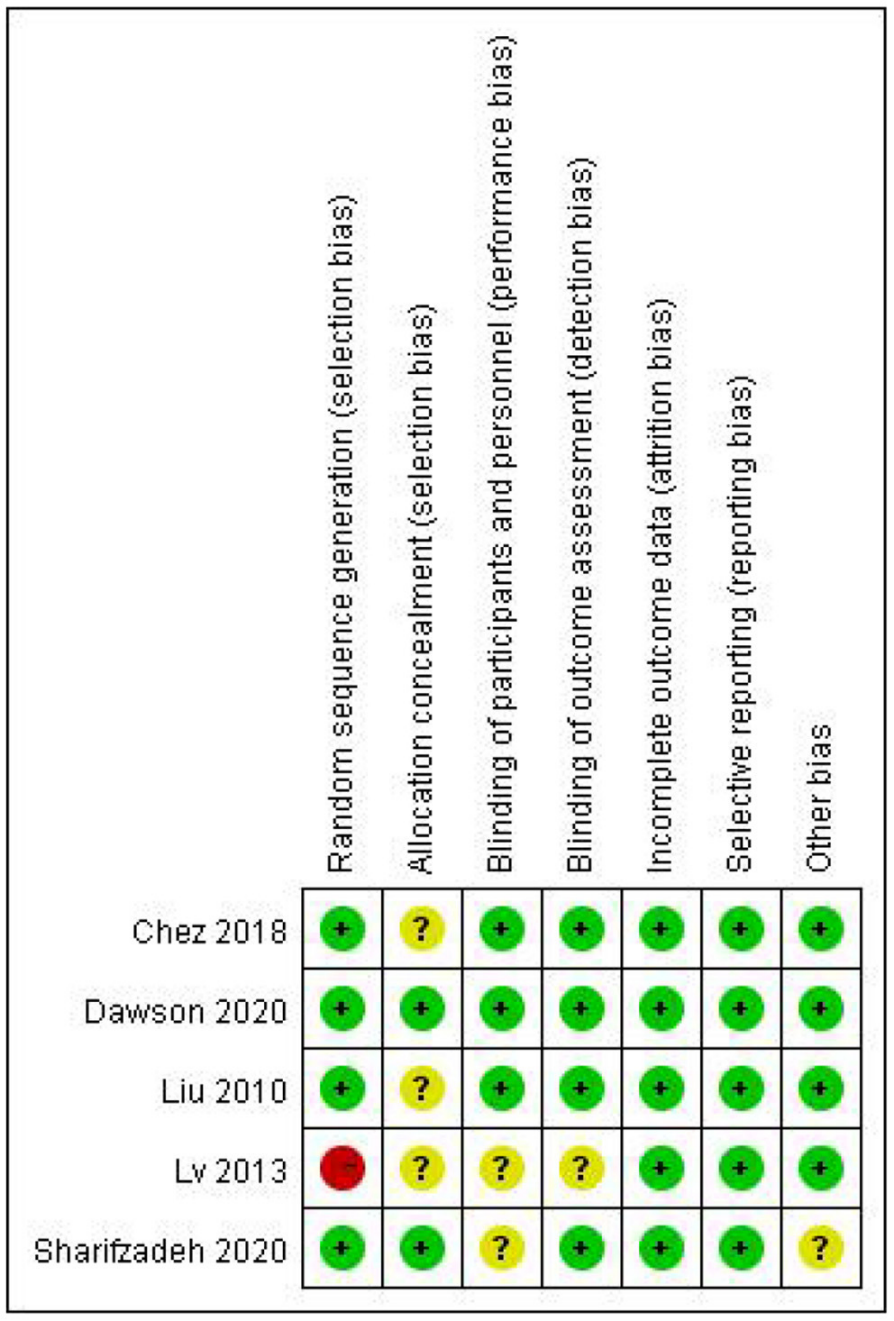
Risk of bias and applicability concerns.

The Figures 2, 3 showed the assessment results of bias risk and methodological suitability of the included studies. Studie by Dawson *et al* was considered high quality and low risk of bias. Lv *et al*. 's study was high risk in the field of random sequence generation and have “Some concerns” in multiple domains that substantially lowers confidence in the result. The other three studies should be considered “Some concerns” according to Cochrane's book in their one or two domains.

### Meta-Analysis

**Figure 4:**
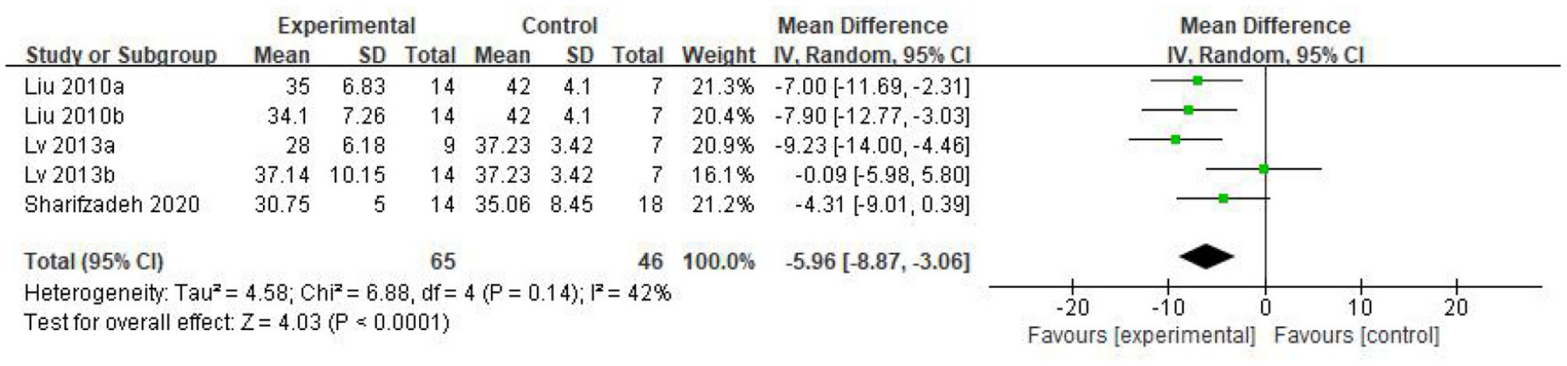
CARS forest plot included in the study.

**Figure 5:**
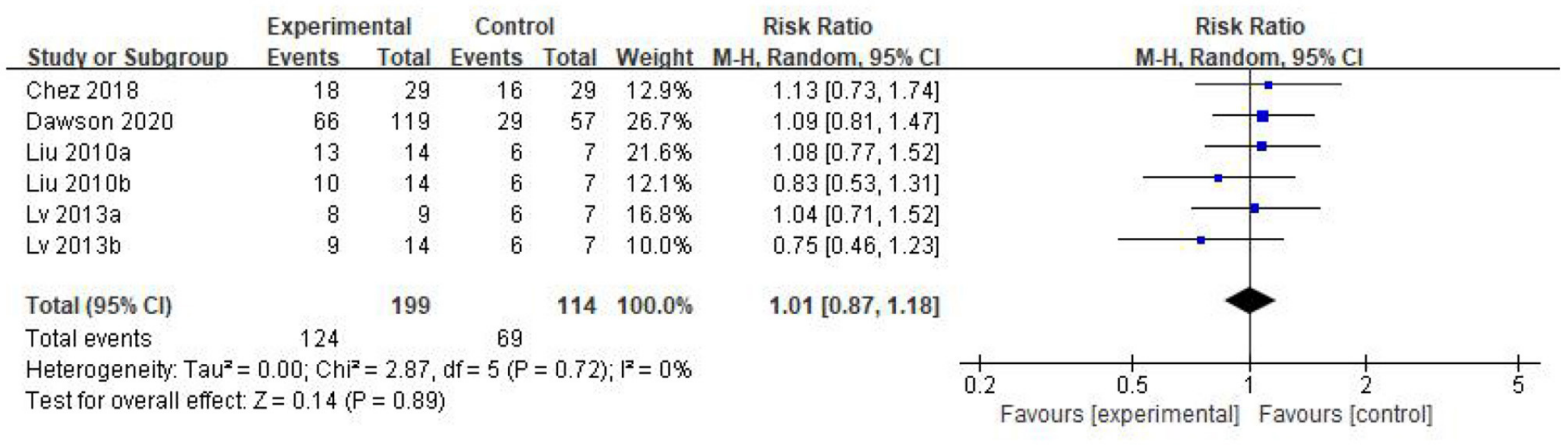
CGI forest plot included in the study.

**Figure 6:**
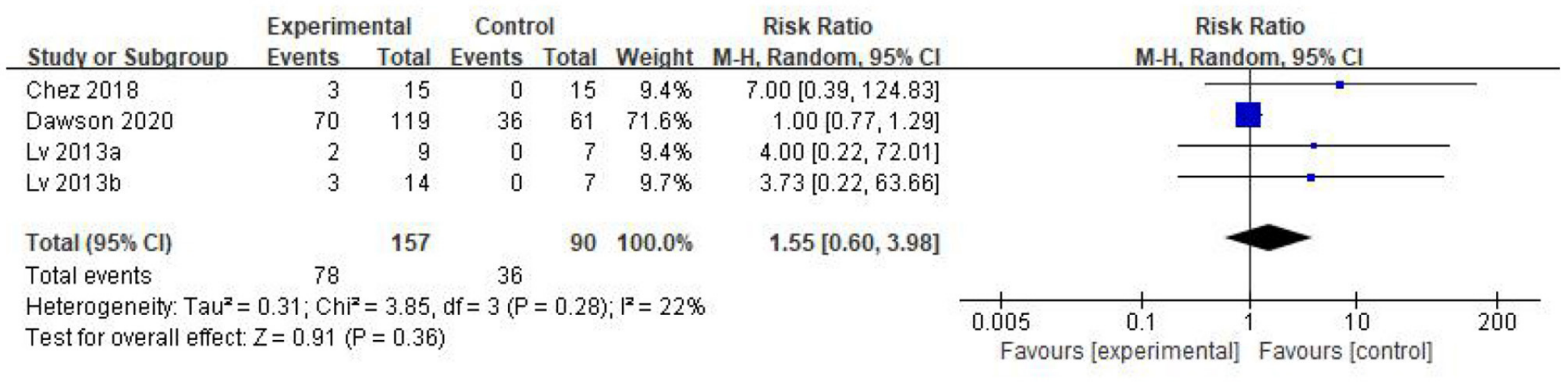
Forest plot of adverse events.

Five eligible articles were meta-analyzed using a random effects model, with CARS (Figure 4) and CGI (Figure 5) as primary and secondary indicators to evaluate the effectiveness of stem cell therapy for autism, and adverse reactions as safety indicator (Figure 6).

As can be seen from the forest diagram in Figure 4, heterogeneity test *p* = 0.14; I^2^ = 42%, indicating moderate heterogeneity. Data showed that the CARS score of the stem cell group was significantly lower than the control group [WMD: −5.96; 95% CI (−8.87, −3.06); *p* < 0.0001] (Figure 4). A higher score of CARS refers to severe disease. Overall, our results showed that the stem cell group had better efficacy in ASD treatment than the control group. In addition, sensitivity analysis was conducted to eliminate outliers in another intervention group (Lv 2013b) of Lv *et al*.'s study and the results were found to be stable (WMD: −7.08; 95%CI [−9.46, −4.70]; *p* < 0.0001; heterogeneity test *p* = 0.53; I^2^ = 0%).

**Figure 7:**
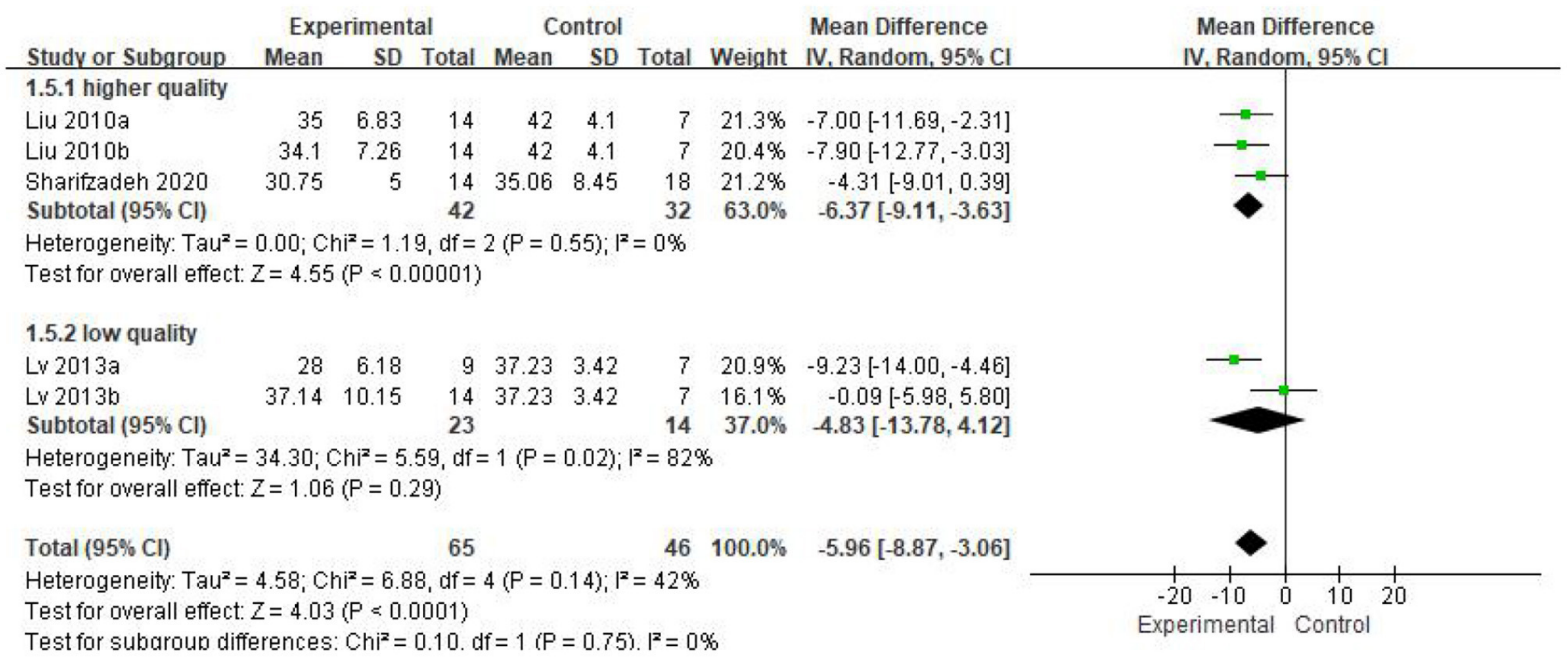
CARS Forest plot of subgroup analysis.

We found that the forest plot of CARS had moderate heterogeneity. According to the results of the methodological quality assessment, we concluded that the research quality of Lv *et al*.'s study was low. Afterward, we conducted a subgroup analysis based on the methodological quality, and found that the combined results of CARS in the high-quality group were stable [WMD: −6.37; 95%CI (−9.11, −3.63); *p* < 0.00001, heterogeneity test *p* = 0.55; I^2^ = 0] (Figure 7). The results of Lv *et al*. 's study showed high heterogeneity and instability in the low-quality group [WMD:−4.83; 95% CI (−13.78, 4.12); *p* = 0.29; heterogeneity test *p* = 0.02; I^2^ = 82%] (Figure 7). Therefore, Lv *et al*.'s study might be the source of heterogeneity. The results in Figure 5 show no significant difference in CGI. Consolidated effect size RR = 1.01, 95%CI [0.87, 1.18], Z = 0.14 (*p* = 0.89), the effective rate for CGI was 62 and 60% in the stem cell group and the control group, respectively. Heterogeneity test I^2^ = 0%, *p* = 0.72, indicating no heterogeneity.

The forest plot in Figure 6 reflects the occurrence events of adverse reactions in each group [RR = 1.55; 95%CI = (0.60, 3.98); *p* = 0.36; Figure 6], there was no significant difference in the incidence of adverse reactions between the stem cell group and the control group, with heterogeneity test *p* = 0.28; I^2^ = 22%. Based on the forest map, we have visual outliers. The sensitivity analysis found that the study of Dawson *et al* was an outlier and the cause of heterogeneity. Whereas, after Dawson *et al*'s study was excluded, the results were more stable [RR = 4.70; 95%CI = (0.90, 24.63); *p* = 0.07; Heterogeneity test *p* = 0.95; I^2^ = 0%].

## Discussion

Stem cells are defined as tissue units of biological systems which is responsible for the regeneration and development of organs and tissues. Stem cells are capable of self-renew and differentiate into multiple cell line ages, therefore, these cells can also be units that evolve through natural selection (28, 29). Hematopoietic stem cells were primarily discovered and used for the treatment of blood-system failure induced by nuclear radiation (30). In recent years, the clinical results show that stem cells have shown promising effects in a variety of chronic and difficult diseases, such as spinal cord injury (31–34), graft-vs. -host disease (GVHD) (35, 36), diabetes and complications (37–39), stroke (40, 41), *etc*. As expected, more and more researchers are attempting to determine the efficacy of stem cell therapy for ASD.

Martínez (26) published the first systematic review and meta-analysis of stem cell therapy for autism in September 2021, but they included uncontrolled studies in the analysis to compensate for the insufficient number of studies. Especially in the studies within the control group, they only extracted data from the intervention group, which might reduce the clinical guiding significance of the conclusion that stem cell therapy significantly improves scales in patients with ASD. Our study demonstrates that stem cell therapy for children with autism appears to be safe and effective. CARS - the primary outcome measurement confirmed the efficacy, whereas, the secondary outcome measurement—CGI, showed no difference between stem cells and control treatment. Furthermore, Bieleninik, Ł (42) found total costs of ASD including health services costs and societal costs, were estimated to be around 2834 EUR in 2 months by analysis with 5 European countries and 4 non-European countries. However, the median total charges and costs for stem-cell transplant hospitalization were $270,198 and $92,717 from 2002 to 2015 (43). Given the persistence of autism, the hospitalization cost also increased dramatically. Therefore, it is extremely important to make the expensive stem cell therapy to gain greatly therapeutic effect. Currently, stem cell treatments for autism is mostly considered a new mean of clinical trials and have just been conducted in only a few places. It is urgent to form the standardized treatment methods and improve the curative effect before that they are popularized in clinical practice.

We can glimpse from the included studies where future autism stem cell therapy needs to be standardized. Firstly, we noted that the doses of cell injections in the included studies were varied. In Sharifzadeh's study (22), subjects received a total of 0.5-1 × 10^8^ cells in the first injection and 0.3-0.5 × 10^8^ cells in the second injection. In other studies (19, 24, 25, 44), subjects received injections ranging from 2 × 10^6^/kg to 2.5 × 10^7^/kg cells at a time. Different doses of cell injection may account for the inconsistent efficacy. Owing to these limited studies, we could not analyze the influence of dosage on the efficacy and safety of stem cell injections. Secondly, there are two ways of cell injection: intravenous injection and intrathecal injection. Intravenous infusion of cells may limit the homing effect, cells could be trapped in organs such as lungs, heart, liver or kidney and get blocked by the blood-brain barrier, which might reduce the therapeutic effect on ASD (45, 46). Furthermore, only two studies were followed up for 12 months, such a short period is not conducive to observing progress in the improvement of core symptoms of autism. Previous studies have suggested improvements observed after 12-month and 18-month follow-up, particularly in the Clinical Global Impression Scale (17, 27, 46). The CGI rating scale has been widely used in psychiatry to evaluate the degree of symptom and therapeutic efficacy, and the Improvement (CGI-I) scale is used to assess the extent a patient has improved or worsened following an intervention, but they are non-ASD specific (47), which might explain why there was no significant difference in the CGI scores between the two groups. Additionally, ASD is a complex neuropsychiatric disorder with substantial phenotypic and genetic heterogeneity (48), reducing the impact of heterogeneity on treatment and evaluation studies is quite important. Moreover, there are multiple sources of stem cells, and the therapeutic effects of stem cells from different sources may vary. Lv (19) and Liu (44) suggest that compared with the control group, the effect of cord blood mononuclear cells (CBMNC) transplantation was significant, however, the combination of CBMNC and umbilical cord mesenchymal stem cells (UCMSCs) had a greater therapeutic effect than CBMNC alone. The small sample size of included studies is also a problem that cannot be ignored. As mentioned above, there are many studies in the field of stem cell therapy for autism that have directly or indirectly demonstrated its effectiveness. However, they did not meet the criteria for inclusion in our analysis and were not meta-analyzed, but their results were equally important.

Overall, the major limitations of the included studies were small sample size, non-specific outcome measures, treatment regimens were not uniform, and lacking adequate follow-up.

## Conclusions

In conclusion, the use of stem cells to treat children with autism may be effective and safe, but we believe that the current evidence is in-sufficient, the conclusions are based on studies that do not have a uniform treatment protocol. Besides, due to the high cost of stem cell therapy and the lack of widespread clinical application, guardians of children with autism need to be discreetly about enrolling in clinical trials of stem cell treatments for autism. It is urgent to establish a standardized treatment protocol through a large number of trials, such as the most suitable stem cell type, administration method and dose need to be screened; the post-treatment evaluation of stem cell therapy need to be improved. These may lead to the discovery of stem cell therapy for autism and its pathogenesis, thus further improving the therapeutic effect. We expect stem cell therapy to be used in the clinical treatment of autism and have significant therapeutic effects, but it is still a lot of work to be done before this can happen.

## Data Availability Statement

The original contributions presented in the study are included in the article/Supplementary Material, further inquiries can be directed to the corresponding author.

## Author Contributions

JQ: conceptualization, writing-reviewing and editing, data extraction, and assessing the risk of bias. ZL: conceptualization, writing-reviewing and editing, data extraction, and assessing the risk of bias. JY: writing-original draft, study selection, research retrieval, and statistical analysis. MZ: study selection, research retrieval, and statistical analysis. LL: study selection, and data extraction. ZZ: writing-reviewing and data extraction. ZH: assessing the quality of studies. LZ: article revision and grammar revision. YL: writing-original draft and statistical analysis. All authors contributed to the article and approved the submitted version.

## Funding

The authors are grateful for the financial support received from the Foundation of Jiangxi Educational Committee (GJJ180791). The Science and Technology Project of Jiangxi Provincial Health Commission (20191079). The Open Project of Key Laboratory of Prevention and treatment of cardiovascular and cerebrovascular diseases, Ministry of Education (XN201913). The Foundation of Technology Innovation Team of Gannan Medical University (TD201806). Key Project Foundation of Gannan Medical University (ZD201831). First Affiliated Hospital of Gannan Medical University, Doctor Start-up Fund (QD076), and Jiangxi Provincial Natural Science Foundation (20212BAB206075).

## Conflict of Interest

The authors declare that the research was conducted in the absence of any commercial or financial relationships that could be construed as a potential conflict of interest.

## Publisher's Note

All claims expressed in this article are solely those of the authors and do not necessarily represent those of their affiliated organizations, or those of the publisher, the editors and the reviewers. Any product that may be evaluated in this article, or claim that may be made by its manufacturer, is not guaranteed or endorsed by the publisher.
