# Supplementary material for: Efficacy and Safety of Stem Cell Therapy in Children With Autism Spectrum Disorders: A Systematic Review and Meta-Analysis
Source: Front Pediatr. 2022 May 4;10:897398. doi: 10.3389/fped.2022.897398 (PMC9114801; doi:10.3389/fped.2022.897398)
Supplement: Supplementary file 1 [file Presentation_1.PDF]

Search Strategy

1. **Pubmed search strategy：**

   Search: ((("Autism Spectrum Disorder"[Mesh]) OR ((((Autism Spectrum Disorders) OR (Autistic Spectrum Disorder)) OR (Autistic Spectrum Disorders)) OR (Disorder, Autistic Spectrum))) AND (("Stem Cells"[Mesh]) OR ((((((((((((((Cell, Stem) OR (Cells, Stem)) OR (Stem Cell)) OR (Progenitor Cells)) OR (Cell, Progenitor)) OR (Cells, Progenitor)) OR (Progenitor Cell)) OR (Mother Cells)) OR (Cell, Mother)) OR (Cells, Mother)) OR (Mother Cell)) OR (Colony-Forming Unit)) OR (Colony Forming Unit)) OR (Colony-Forming Units)) OR (Colony Forming Units)))) AND (randomized controlled trial[Publication Type] OR randomized[Title/Abstract] OR placebo[Title/Abstract])

2. **Cochrane search strategy：**

   Search Name: Cochrane-ASD-hucmsc
   Date Run:24/07/2021 02:39:53
   Comment:

   ID Search Hits
   #1 (Autism Spectrum Disorders):ti,ab,kw OR (Autistic Spectrum Disorder):ti,ab,kw OR (Autistic Spectrum Disorders):ti,ab,kw OR (Disorder, Autistic Spectrum):ti,ab,kw (Word variations have been searched) 2534
   #2 MeSH descriptor: [Stem Cells] explode all trees 816
   #3 (Cell, Stem):ti,ab,kw OR (Cells, Stem):ti,ab,kw OR (Stem Cell):ti,ab,kw OR (Progenitor Cells):ti,ab,kw OR (Cell, Progenitor):ti,ab,kw (Word variations have been searched) 14486
   #4 (Cells, Progenitor):ti,ab,kw OR (Progenitor Cell):ti,ab,kw OR (Mother Cells):ti,ab,kw OR (Cell, Mother):ti,ab,kw OR (Cells, Mother):ti,ab,kw (Word variations have been searched) 3007
   #5 (Mother Cell):ti,ab,kw OR (Colony-Forming Unit):ti,ab,kw OR (Colony Forming Unit):ti,ab,kw OR (Colony-Forming Units):ti,ab,kw OR (Colony Forming Units):ti,ab,kw (Word variations have been searched) 3264
   #6 #2 OR #3 OR #4 OR #5 17582
   #7 MeSH descriptor: [Autism Spectrum Disorder] explode all trees 1582
   #8 #7 OR #1 3056
   #9 #6 AND #8 7

3. **Embase search strategy**

   Session Results
   ....
   No. Query Results Results Date
   #26. #7 AND #24 AND #25 36 24 Jul 2021
   #25. 'random' OR 'placebo' OR 'double-blind' 935,045 24 Jul 2021
   #24. #8 OR #9 OR #10 OR #11 OR #12 OR #13 OR #14 OR #15 OR #16 OR #17 OR #18 OR #19 OR #20 OR #21 OR 534,643 24 Jul 2021

#22 OR #23

#23.	'colony forming units'	19,169	24 Jul 2021
#22.	'colony-forming units'	19,169	24 Jul 2021
#21.	'colony forming unit'	47,706	24 Jul 2021
#20.	'colony-forming unit'	47,706	24 Jul 2021
#19.	'mother cell'	1,678	24 Jul 2021
#18.	'cells, mother'	13	24 Jul 2021
#17.	'cell, mother'	15	24 Jul 2021
#16.	'mother cells'	1,196	24 Jul 2021
#15.	'progenitor cell'	41,147	24 Jul 2021
#14.	'cells, progenitor'	306	24 Jul 2021
#13.	'cell, progenitor'	986	24 Jul 2021
#12.	'progenitor cells'	80,159	24 Jul 2021
#11.	'stem cells'	286,467	24 Jul 2021
#10.	'cells, stem'	1,062	24 Jul 2021
#9.	'cell, stem'	3,566	24 Jul 2021
#8.	'stem cell'/exp	406,043	24 Jul 2021
#7.	#1 OR #2 OR #3 OR #4 OR #5 OR #6	83,697	24 Jul 2021
#6.	'disorder, autistic spectrum'	24	24 Jul 2021
#5.	'autistic spectrum disorders'	1,214	24 Jul 2021
#4.	'autistic spectrum disorder'	1,430	24 Jul 2021
#3.	'autism spectrum disorders'	16,192	24 Jul 2021
#2.	'autism spectrum disorder'	25,759	24 Jul 2021
#1.	'autism'/exp	80,679	24 Jul 2021

4. **Web of science search strategy**

TS=(Autism Spectrum Disorder OR Autism Spectrum Disorders OR Autistic Spectrum Disorder OR Autistic Spectrum Disorders OR Disorder, Autistic Spectrum)

TS=(Stem Cells OR Cell, Stem OR Cells, Stem OR Stem Cell OR Progenitor Cells OR Cell, Progenitor OR Cells, Progenitor OR Progenitor Cell OR Mother Cells OR Cell, Mother OR Cells, Mother OR Mother Cell OR Colony-Forming Unit OR Colony Forming Unit OR Colony-Forming Units OR Colony Forming Units)

TS=(randomized controlled trial OR randomized OR placebo)
